# Persistent Fever in Tuberculosis: Clinical Experience and Literature Review

**DOI:** 10.7759/cureus.69391

**Published:** 2024-09-14

**Authors:** Bereket Tewoldemedhin, Saif Al-Ethawi, Wassim Abouzeid, Charity Iheagwara, Maria Szabela, Jack Boghossian, Jihad Slim

**Affiliations:** 1 Internal Medicine, Suburban Community Hospital (Lower Bucks Hospital), Bristol, USA; 2 Infectious Diseases, Saint Michael's Medical Center, Newark, USA; 3 Internal Medicine, Saint Michael's Medical Center, Newark, USA

**Keywords:** fever and tuberculosis, immune reconstitution syndrome, persistent fever, steroids in tuberculosis, tb – tuberculosis

## Abstract

Fever in tuberculosis has always been a challenge to clinicians treating the disease throughout history. There is a constant interplay between the host immune response and the bacillary load that results in high-grade fevers in patients with tuberculosis. In the setting of pulmonary tuberculosis, there is scant data regarding how long fevers last and the exact pathophysiology of its prolonged duration, especially once appropriate antituberculosis medication is initiated.

This case report elucidates our experience in treating a patient presenting with smear-positive pulmonary tuberculosis with significant bacillary load; despite responding microbiologically to antituberculosis therapy, he clinically continued to have persistent fever.

## Introduction

*Mycobacterium tuberculosis* (*M. tuberculosis*) infection begins by inhalation of aerosolized droplets that contain the bacilli by a susceptible host that has previously been unaffected, resulting in the deposition of the organism within the lungs [1,2]. This initial process has various possible outcomes with the best and the rarest clinical scenario being the clearance of the organism by the adaptive immune response [1,2]. Another outcome is the containment of the viable organisms by the immune system orchestrated by the cell-mediated immune response; this process results in latent tuberculosis [2]. This accounts for 90% of outcomes in individuals with intact immunity [2,3]. Various factors, including the host immune system and pathogenicity of *M. tuberculosis,* are important in determining this outcome [1-3]. The tuberculosis bacilli proliferate within alveolar macrophages, inciting a complex immunologic response with the production of cytokines resulting in chemotaxis of inflammatory cells and granulomatous tubercle formation [1,2]. Reactivation of the disease then occurs after a period of clinical latency among individuals who develop immunosuppression [1,2]. The granulomatous response, which is the aggregate of macrophages, T lymphocytes, and giant cells, results in a relative immunosuppressive state [2,3]. This occurs as a result of the presence of regulatory T cells that constantly produce IL-10 and TNF-beta, impairing the cell-mediated immune response within the granuloma and restricting the lysis of infected macrophages. This relative immunosuppressive state allows* M. tuberculosis* bacilli to continue to survive within the granuloma state [2,3]. Any disruption in the immune status of the patient including nutritional, viral infection, or increments in metabolic demand can result in reactivation of the bacilli. This can manifest as localized or disseminated disease based on the degree of immunosuppression of the individuals [3]. Fever is the most common symptom worldwide, accounting for 70% of tuberculosis patients [2-4]. Fever onset is generally gradual and can range from low- to high-grade, lasting an average of two to three weeks [2,3]. 

Prolonged fever lasting more than three weeks in pulmonary tuberculosis patients on appropriate antituberculosis medication is a diagnostic challenge that often includes a long list of possible etiologies, including superimposed infections, adverse drug reactions, potentially resistant *M. tuberculosis* bacilli, and complicated tuberculosis disease itself [3,4]. In rare circumstances, a thorough diagnostic workup could be inconclusive, often presenting diagnostic and therapeutic challenges to the clinicians treating these patients. The place of adjunct steroids in such cases is still poorly understood and a topic of constant debate. Whether corticosteroids could be used as part of the host-directed therapy in these patients remains to be seen. In this case report we present our clinical experiences in managing this particular challenging scenario. This case report will help in clinical decision-making and serve as a benchmark for future research on the use of steroids in these complex situations. 

## Case presentation

A 25-year-old male presented to the emergency department with a complaint of a one-month history of cough, fever, and sore throat. He had gone to urgent care at the outset of his symptoms and was treated with oral amoxicillin for seven days for presumed pharyngitis with minimal improvement. He continued to have high-grade fevers that particularly worsened at night, with generalized malaise and drenching night sweats. He started to have a non-productive cough for the same period with pleuritic right-sided chest pain. A review of systems was positive for weight loss of approximately 13 lbs in one month, along with anorexia and malaise. 

The patient had immigrated from Guatemala to the United States in 2012. He denied having any health issues prior to his current presentation. He had not traveled back since 2012. He had quit smoking three years prior after a six-pack-year smoking history. He had no allergies, no exposure to sick persons, and did not have any pets. 

His vital signs were as follows: temperature 100.7 °F, heart rate 100 beats/minute, blood pressure 107/66 mmHg, respiratory rate 24 breaths/minute, oxygen saturation 94% on room air. The physical exam revealed a slender physique with no lymphadenopathy detected. The cardiovascular exam was unremarkable except for mild tachycardia. The pulmonary exam was significant for posterior bilateral course bronchial breath sounds with coarse rales on the upper third of the lung fields. There was no skin ulceration or discharge present. Laboratory results showed a normal leukocyte count with mild thrombocytosis and elevated acute phase reactants. Lactate dehydrogenase was elevated. The complete metabolic panel was unremarkable except for mildly increased alkaline phosphate levels (Table 1).

**Table 1:** Laboratory test results of the patient

Parameters	Results	Reference values
Hemoglobin	11.3 g/dl	12-16 g/dl
Platelets	540,000/µL	150,000-400,000/µL
White blood cells	8100/µL	3100-10,500/µL
Neutrophils	4200/µL	1500-7000/µL
Neutrophils (relative percent)	78.9%	40-60%
Lymphocytes	900/µL	1000-4000/µL
Lymphocytes (relative percent)	14.2%	20-40%
Monocytes	500/µL	300-900/µL
Monocytes (relative percent)	9.0%	4-8%
Lactate dehydrogenase	413 IU/L	120-245 IU/L
Erythrocyte sedimentation rate	60 mm/hour	0-20 mm/ hour
C-reactive protein	9.8mg/dl	0.0-0.8 mg/dL
Glucose	88 mg/dl	70-140 mg/dl
Blood urea nitrogen	14.0 mg/dl	6.0-24 mg/dl
Creatinine	0.52 mg/dl	0.5-1.0 mg/dl
Sodium	139 mmol/L	136-145 mmol/L
Potassium	4.0 mmol/L	3.5-5.3 mmol/L
Chloride	107 mmol/L	98-110 mmol/L
Albumin	3.7 g/dl	3.6-5.1 g/dl
Total protein	6.7 g/dl	6.4-8.4 g/dl
Total bilirubin	0.3 g/dl	0.2-1.2 g/dl
Aspartate transferase (AST)	14 U/L	10-36 U/L
Alanine transaminase (ALT)	16 U/L	10-49 U/L
Alkaline phosphatase (ALP)	128 U/L	46-116 U/L

**Figure 1:**
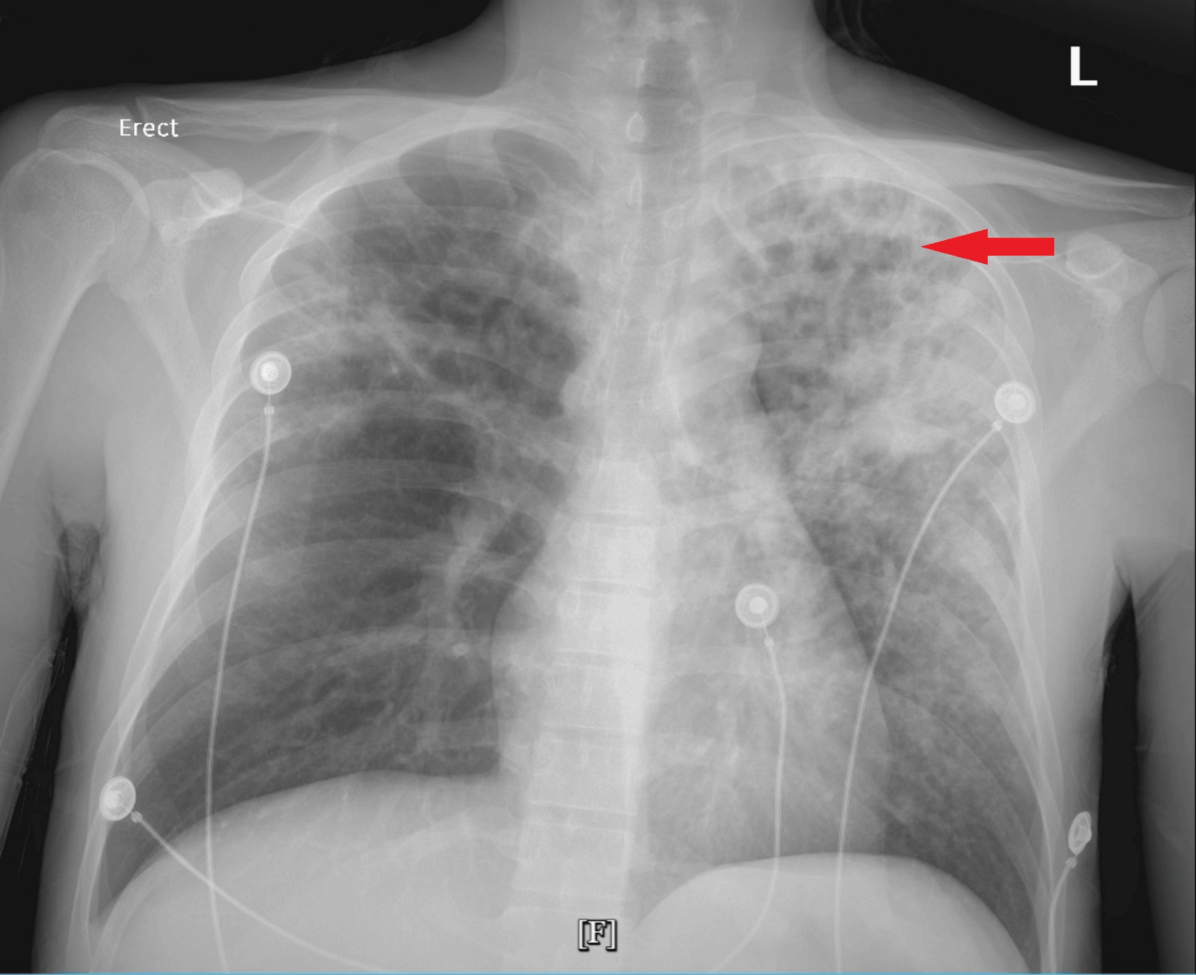
Chest x-ray showing patchy consolidation with cavitation and fibrosis in the left upper lung zone (red arrow)

Tests for other pathogens including HIV, COVID-19, malaria, dengue, and typhoid revealed negative results. The coagulation panel and thyroid function tests were unremarkable. Serology titers for Epstein-Barr virus and cytomegalovirus were negative. A chest X-ray revealed multifocal areas of patchy consolidation in both lung fields, more prominent in the upper- to mid-lung zones of the left lung compared to the right. Patchy areas of fibrosis and possible cavitation were seen in the left upper zone. Left apical pleural thickening was also noted (Figure 1). 

**Figure 2:**
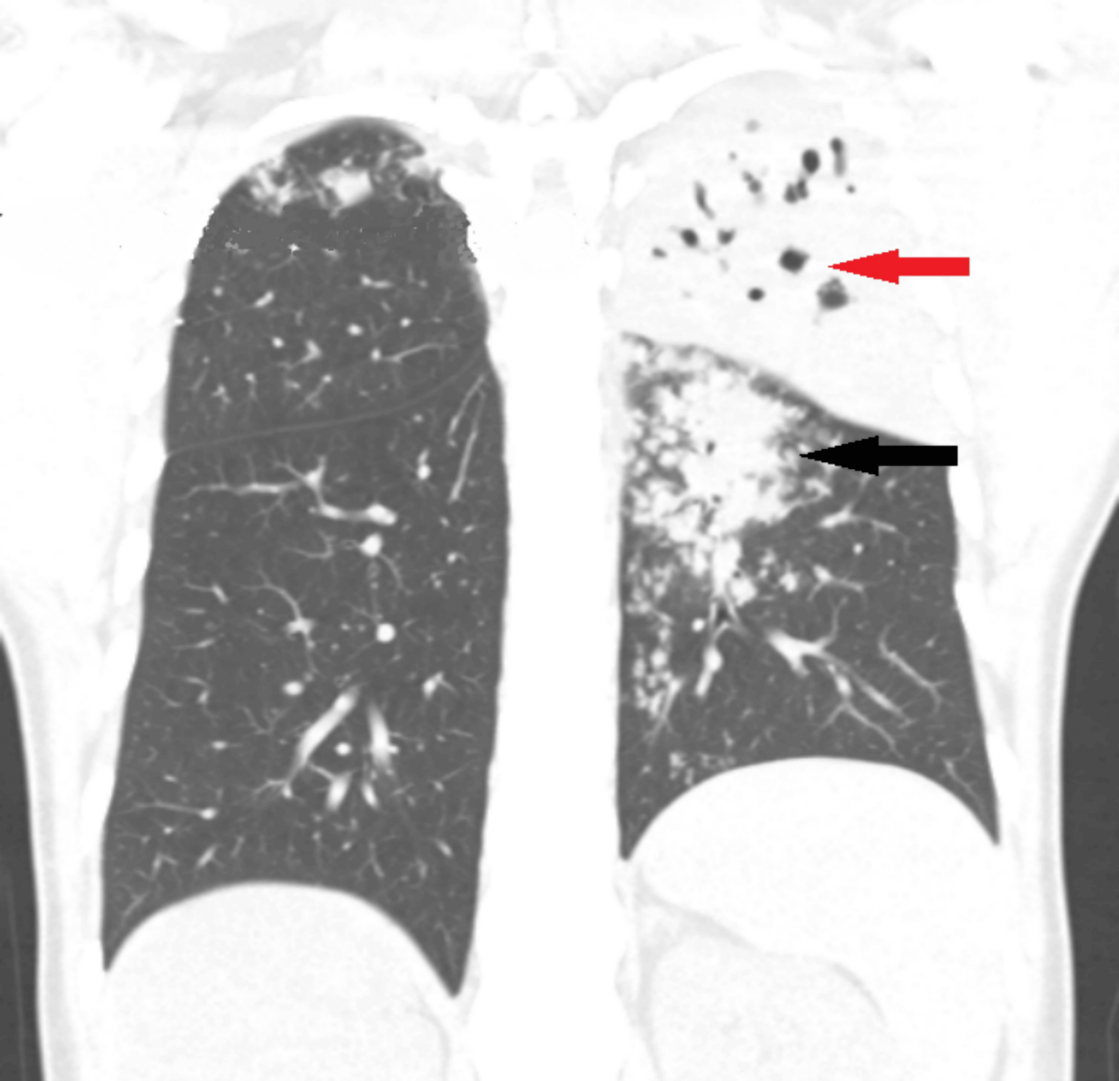
Computer tomography scan of the chest with coronal section showing large left apico-posterior consolidation patch with cavitary changes (red arrow) and left superior lingual segment consolidation with tree in bud pattern (black arrow).

A computer tomography (CT) scan of the chest showed a large consolidation patch affecting the apico-posterior and superior lingular segments of the left upper lobe with multiple small areas of cavitary and bronchiectatic changes (Figure 2).

**Figure 3:**
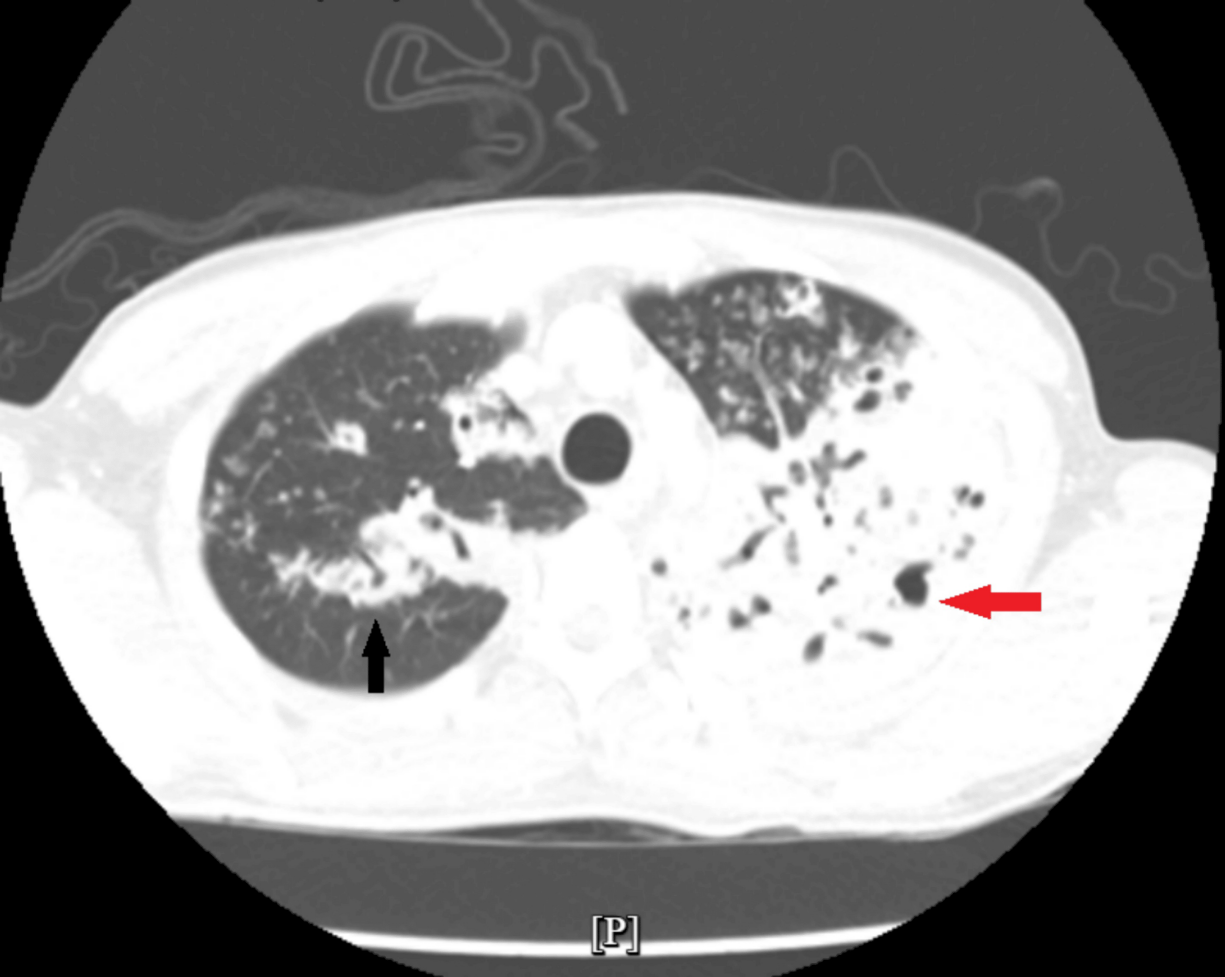
Computer tomography scan of the chest with axial section showing multifocal consolidation with cavitation on the left upper lobe (red arrow) and patchy consolidation with tree in bud and cavitation on the right upper lobe (black arrow)

An axial reconstruction view of the CT scan showed smaller multifocal patchy consolidations in the bilateral lungs with cavitary changes and multiple centrilobular nodules exhibiting tree-in-bud patterns, notably in the superior segment of the left lower lobe, anterior and apical segment of the right upper lobe (Figure 3).

**Figure 4:**
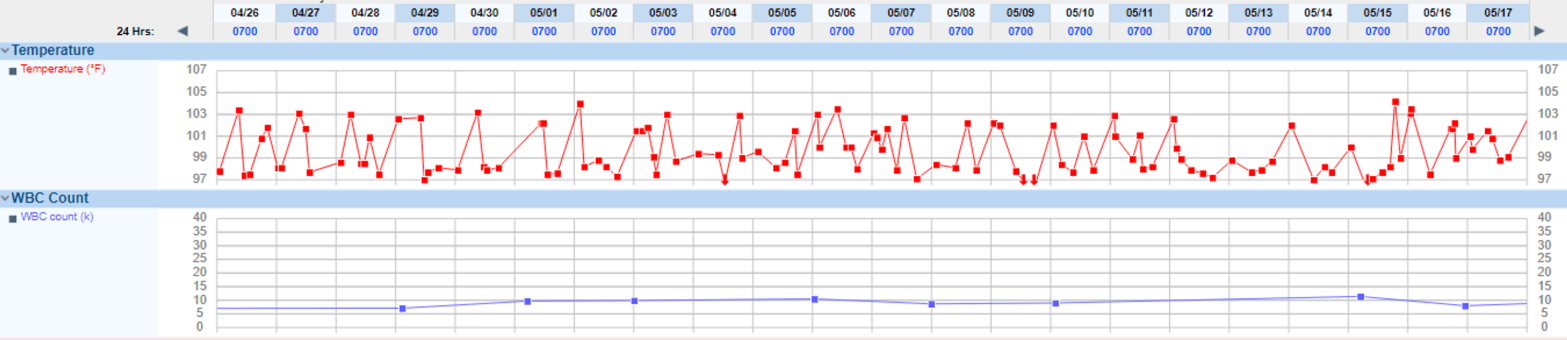
Temperature trend with persistent night time spikes of high grade fever

Acid-fast staining of induced sputum yielded 3+ acid-fast bacilli via the direct Ziel Neilson method. *M. tuberculosis* was detected on PCR with rifampin sensitivity. The patient had been on airborne isolation precautions from the time of his presentation. After the acid-fast stain results, he was initiated on weight-based rifampin 600 mg daily, isoniazid 300 mg daily, pyrazinamide 1500 mg daily, and ethambutol 1200 mg daily (RIPE) therapy with daily pyridoxine 50 mg daily for the assessment of smear-positive pulmonary tuberculosis with extensive cavitating lesions. He continued to have nighttime fevers throughout his stay; his temperature curve showed spikes reaching 103°F on most nights (Figure 4).

The patient's sputum acid-fast bacilli load continued to decrease from 3+ to 2+ within two weeks of antituberculosis therapy initiation, then to 1+ within four weeks. Sputum smears became sterile with no acid-fast bacilli after six weeks of therapy; however, he continued to have fevers. Full septic workup and multiple blood cultures were negative. His complete metabolic panel as well as complete blood count remained relatively unchanged from his presentation. There was no significant increment or decrease in his transaminases or alkaline phosphatase levels; there was also no change in the white blood cell count throughout his stay. The erythrocyte sedimentation rate declined marginally from 60 mm/hr to 40 mm/hr and stabilized. C-reactive protein marginally trended down from 9.8 to a nadir of 5 and remained at that level. Chest X-ray was repeated every week with no interval change in his pulmonary findings. Chest CT scans were repeated at two weeks and at four weeks, with no significant interval change observed. A CT scan of the abdomen and pelvis revealed no lesions in the intra-abdominal region, psoas muscle, or retroperitoneal areas.

The susceptibility pattern of *M. tuberculosis* showed a pan-susceptible organism that was susceptible to isoniazid at 0.1 µg/ml, rifampin at 1 µg/ml, streptomycin at 1 µg/ml, ethambutol at 5 µg/ml, and pyrazinamide at 100mg/ml. Levels of isoniazid and rifampin were checked and reported to be within therapeutic level. Fungal cultures and serum beta D glucan levels were negative. Autoimmune workup to rule out drug-induced lupus including anti-histone antibody, anti-dsDNA antibody, and ANA panel was negative. Drug levels for the antituberculosis medication were checked and found to be within therapeutic levels. 

At four weeks of persistent fever episodes, idiosyncratic drug-induced fever needed to be ruled out and hence isoniazid was discontinued for three days with no change in the fever pattern. All medications were stopped to evaluate the effect on the fever curve and the patient continued to have high-grade fevers at night. Antituberculosis medications were reinstated after 72 hours. Adrenocortical suppression was ruled out by checking the morning cortisol levels as well as the cosyntropin test, both of which were within their normal ranges of 19.69µg/dL and 26µg/dL at 30 minutes, respectively. 

After much deliberation, the patient was initiated on prednisone 40 mg daily which was added to his regimen of rifampin, isoniazid, pyrazinamide, and ethambutol (RIPE). The patient continued to respond very well to the regimen. The patient defervesced within four days of initiation of prednisone. The prednisone was tapered down over the next two weeks. He completed the remainder of the direct observed therapy of the antituberculosis medication without further complications. He has subsequently entered the continuation phase with rifampin and isoniazid. The patient continued to tolerate the regimen without further complications or episodes of fever.

## Discussion

Fever is a significant symptom in patients with active pulmonary tuberculosis [1]. Following the inhalation of aerosolized droplets containing *M. tuberculosis* that an infected individual expectorates, these droplets travel through the respiratory tract and become entrapped within the mucus-secreting goblet cells of the upper airway [1, 2]. The bacilli that bypass this entrapment reach the aerated lower portions of the lungs where they activate the innate immune system, namely the alveolar macrophages. The macrophages engulf the infecting bacilli and attempt to destroy it by releasing proteolytic enzymes and cytokines, including tumor necrosis factor-alpha and interferon-gamma [2]. This reaction signals the migration of T lymphocytes towards the active inflammatory site with a resultant cell-mediated immune response culminating in granuloma formation [2]. This process results in the initial hyperpyrexia and fever associated with tuberculosis and is seen in 60-85% of patients with pulmonary tuberculosis [1,2]. The concomitant inflammatory process results in septic decay of damaged bronchi and the absorption of toxins from the pulmonary cavities, which contributes to the fever in the early stage of the disease [1,2]. The presence of constant pyrogens from the infectious process causes an increase in the thermoregulatory set point of the hypothalamus, culminating in the high-grade fever seen in tuberculosis [1-3]. There are various theories to explain the distinct circadian rhythm and night sweats observed in TB [1]. This has been postulated to be due to the reduction in serum cortisol levels that happen during the night, which is responsible for modulating both innate and acquired immune responses, and in doing so also suppresses fever [1,2]. The decreased secretion of this hormone at night in turn contributes to the typical fevers and night sweats observed with tuberculosis [1,2]. 

Fever itself is a high catabolic state that exerts an increased metabolic demand on the body, affecting the patient's recovery; it should be addressed if prolonged. It can induce multiple pathophysiological changes with deleterious effects on the body including direct cellular damage with a disruption of cell membrane stability and denaturing of proteins vital for cellular function within the body [3,4]. There is further increased free radical production within the body and oxidative stress, worsening the inflammatory cascade within the body and significantly increasing mortality in patients [3]. 

The estimates of the duration of fever in patients with pulmonary tuberculosis are mostly from observational studies [3,4]. One study that involved 80 patients with bacteriologically proven smear-positive pulmonary tuberculosis showed that with appropriate antituberculosis medication initiation, the average duration of fever lasted approximately five days to 21 days, with a median of 8-10 days [4,5]. Another study trying to identify underlying factors for patients experiencing prolonged fevers while on therapy for pulmonary tuberculosis found that the most identified causes included superimposed infection in 32.5%, drug fever in 22.5%, direct complications of tuberculosis in 22.5%, with more extensive disease including empyema and abscesses in the paraspinal or periarticular regions. Hypersensitivity reaction to the bacilli in 12.5%, and drug resistance in 10% were the remainder of the identified causes [4-6]. 

The use of steroids in isolated pulmonary tuberculosis has always been a contentious subject. In patients with acute hypoxic respiratory failure from severe tuberculosis, using corticosteroids had no significant reduction in the 90-day mortality rate in patients who were critically ill [7-9]. In a particular subset of patients with pulmonary tuberculosis with persistent fevers, one study involved a retrospective review of data of patients from a tertiary inpatient care center who were sputum smear-positive pulmonary tuberculosis with high-grade fevers of 39.6 °C on average [9-12]. The patients were all on effective antituberculosis therapy which was confirmed by the reduction in their bacillary load. After all other causes of fever were excluded, the patients were placed on 20-60 mg of prednisone daily until temperature normalized. The study showed that with the initiation of oral prednisone, there was a reduction in the mean temperature from 39.6-38.1 °C in all patients within 24 hours. All the patients in the study had clinical responses with defervescence within five to seven days [10,11,13]. The duration of prednisone therapy was continued for a mean of 20 days and tapered off with no side effects noted on the patients [13-15]. To date, besides this specific retrospective data review, there is no other reported evidence or other randomized control trials showing the efficacy of steroids in treating fever in pulmonary tuberculosis patients who continue to be persistently febrile despite effective antituberculosis therapy [13,15,16]. 

Our patient had a clinical background similar to the above subset and also exhibited a good bacteriologic response to antituberculosis treatment with persistent high-grade fevers that risked a high catabolic state along with significant morbidity and mortality; additionally, his treatment improved prednisone as well.

## Conclusions

Fever is one of the greatest threats to humanity and should not be taken lightly. In patients with pulmonary tuberculosis, the pathophysiology of fever is multifactorial and involves a complex interplay between the immune system and the bacilli themselves. If fever persists for more than three weeks, underlying etiologies should be thoroughly investigated; once all other causes have been ruled out, a trial of steroid therapy can be considered as a therapeutic option. A thorough workup and vigilance to address persistent high-grade fevers in these patients can significantly reduce morbidity and mortality associated with the detrimental catabolic and physiologic effects of prolonged fever in addition to the infectious process. Further studies are needed to assess the underlying long-term benefits of using steroids in these patients and this case report serves as a benchmark. 

## References

[1] Luies L, du Preez I (2020). The echo of pulmonary tuberculosis: mechanisms of clinical symptoms and other disease-induced systemic complications. Clin Microbiol Rev.

[2] Rosha D (2002). Prolonged fever during the treatment of pulmonary tuberculosis. Med J Armed Forces India.

[3] Walter EJ, Hanna-Jumma S, Carraretto M, Forni L (2016). The pathophysiological basis and consequences of fever. Crit Care.

[4] Berger HW, Rosenbaum I (1968). Prolonged fever in patients treated for tuberculosis. Am Rev Respir Dis.

[5] Barnes PF, Chan LS, Wong SF (1987). The course of fever during treatment of pulmonary tuberculosis. Tubercle.

[6] Tsao TC, Tsai YH, Lan RS, Shieh WB, Lee CH (1989). Fever characteristics in tuberculosis--clinical observation in 597 cases. Changgeng Yi Xue Za Zhi.

[7] Kiblawi SS, Jay SJ, Stonehill RB, Norton J (1981). Fever response of patients on therapy for pulmonary tuberculosis. Am Rev Respir Dis.

[8] Bahurupe S, Dhote S, Phatak S, Mitra K, Onkar P (2023). Liver tuberculosis presenting as fever of unknown origin: a case report and imaging spectrum with a review of literature. Cureus.

[9] Mohammad A, Haider V, Islam M, Nashwan AJ, Al Hariri B (2023). An unusual case of disseminated tuberculosis in a healthy adult: A case report. Medical Reports.

[10] Barakat MT, Scott J, Hughes JM (1996). Grand rounds--Hammersmith Hospital. Persistent fever in pulmonary tuberculosis. BMJ.

[11] Antoniou M, Chloros D, Spyratos D, Giouleka P, Sichletidis L (2011). Therapeutic pneumoperitoneum in a patient with pulmonary tuberculosis and persistent fever. BMJ Case Rep.

[12] Clarke P, Allen MB (1996). Pulmonary tuberculosis and steroids. Chest.

[13] Critchley JA, Orton LC, Pearson F (2014). Adjunctive steroid therapy for managing pulmonary tuberculosis. Cochrane Database Syst Rev.

[14] Shubin H, Lambert RE, Heiken CA, Sokmensuer A, Glaskin A (1959). Steroid therapy and tuberculosis. J Am Med Assoc.

[15] Yang JY, Han M, Koh Y (2016). Effects of corticosteroids on critically ill pulmonary tuberculosis patients with acute respiratory failure: a propensity analysis of mortality. Clin Infect Dis.

[16] Muthuswamy P, Hu TC, Carasso B, Antonio M, Dandamudi N (1995). Prednisone as adjunctive therapy in the management of pulmonary tuberculosis. Report of 12 cases and review of the literature. Chest.

